# Current insights and advances into plant male sterility: new precision breeding technology based on genome editing applications

**DOI:** 10.3389/fpls.2023.1223861

**Published:** 2023-07-13

**Authors:** Silvia Farinati, Samela Draga, Angelo Betto, Fabio Palumbo, Alessandro Vannozzi, Margherita Lucchin, Gianni Barcaccia

**Affiliations:** Department of Agronomy, Food, Natural Resources, Animals and Environment (DAFNAE), University of Padova, Legnaro, PD, Italy

**Keywords:** male sterility, precision breeding, genome editing, CRISPR/Cas system, DNA free, transgenerational gene editing, food crops, ornamental species

## Abstract

Plant male sterility (MS) represents the inability of the plant to generate functional anthers, pollen, or male gametes. Developing MS lines represents one of the most important challenges in plant breeding programs, since the establishment of MS lines is a major goal in F1 hybrid production. For these reasons, MS lines have been developed in several species of economic interest, particularly in horticultural crops and ornamental plants. Over the years, MS has been accomplished through many different techniques ranging from approaches based on cross-mediated conventional breeding methods, to advanced devices based on knowledge of genetics and genomics to the most advanced molecular technologies based on genome editing (GE). GE methods, in particular gene knockout mediated by CRISPR/Cas-related tools, have resulted in flexible and successful strategic ideas used to alter the function of key genes, regulating numerous biological processes including MS. These precision breeding technologies are less time-consuming and can accelerate the creation of new genetic variability with the accumulation of favorable alleles, able to dramatically change the biological process and resulting in a potential efficiency of cultivar development bypassing sexual crosses. The main goal of this manuscript is to provide a general overview of insights and advances into plant male sterility, focusing the attention on the recent new breeding GE-based applications capable of inducing MS by targeting specific nuclear genic loci. A summary of the mechanisms underlying the recent CRISPR technology and relative success applications are described for the main crop and ornamental species. The future challenges and new potential applications of CRISPR/Cas systems in MS mutant production and other potential opportunities will be discussed, as generating CRISPR-edited DNA-free by transient transformation system and transgenerational gene editing for introducing desirable alleles and for precision breeding strategies.

## 1 Introduction

Plant male sterility (MS) refers to the inability of the plant to generate functional anthers, pollen, or male gametes, although female fertility remains unaffected (Kaul, 1988). Therefore, male sterile plants cannot undergo self-pollination, but they can be fertilized by male fertile plants. The establishment of MS lines is a major goal in F_1_ hybrid production and marketing because by disabling self-fertilization, it is possible to facilitate the exploitation of heterosis in predominantly autogamous species (Longin et al., 2012; Kim and Zhang, 2018; Li et al., 2022; Ramlal et al., 2022). In the past, the main way to avoid considerable shares of progeny derived from self-pollination, even in species with predominantly allogamous fertilization, was to perform physical emasculation with chemical, mechanical or even manual methods. The main advantage in the use of MS lines is the reduction of costs, time and energy related to these emasculation procedures (Colombo and Galmarini, 2017). For these reasons, MS lines have been developed in several species of economic interest (Abbas et al., 2021; Wang et al., 2023), particularly in horticultural crops and ornamental plants (Yamagishi and Bhat, 2014; Barcaccia et al., 2016; Khan and Isshiki, 2016; Jindal et al., 2019; Singh and Khar, 2021). MS exhibits, in most cases, Mendelian inheritance, which is controlled either by the coordinated action of nuclear and cytoplasmatic genes or exclusively by nuclear genes (Kaul, 1988). The first scenario, defined as *cytoplasmic male sterility* (CMS), or three-line breeding system, relies on loci localized within the mitochondrial genome (Rogers and Edwardson, 1952; Chen and Liu, 2014). Cytoplasmic maternal inheritance causes all progeny derived from plants carrying the S locus (*sterile*) cytoplasm to inherit the male sterility trait (Budar and Pelletier, 2001; Yamagishi and Bhat, 2014; Xu et al., 2022). This condition can be overcome by nuclear genes that are functional in dominant conditions (Jindal et al., 2019); they are defined as *restorers of fertility* (*Rf*) and can suppress or downregulate the CMS genes and revert male sterility (Schnable and Wise, 1998; Ning et al., 2020). In contrast*, genic male sterility* (GMS), also reported as *nuclear male sterility* (NMS), or two-lines breeding system, is generally controlled by single nuclear genes, mostly by recessive alleles (*ms*) (Colombo and Galmarini, 2017; Manjunathagowda, 2021).

Although genes involved in MS have not yet been characterized in many species, the complex molecular mechanisms at the bases of GMS and CMS have been studied in the most important crops at the transcriptomic, biochemical and epigenetic levels (Fan et al., 2016; Li et al., 2019; Liu et al., 2022; Nie et al., 2023). Potential limitations in the application of MS systems in agricultural species reside first in the availability of MS resources, in the difficulty encountered when introgressing the trait into commercial varieties (e.g., linkage drag issues), and in the maintenance of the MS lines. Furthermore, considering that MS is detectable only during flowering stages, selecting plants characterized by GMS systems may be a challenge for preventing self-pollination. In addition, MS can be influenced by environmental conditions, resulting in instability and being a major issue for conducting crosses. Nevertheless, external conditions have been successfully exploited in rice and wheat, manipulating temperature or photoperiod to guarantee alternating cross-pollination or self-pollination (*environmental genic male sterility* – EGMS) (Zhou et al., 2012; Meng et al., 2016).

Developing MS lines therefore represents one of the most important challenges in plant breeding programs. Over the years, MS has been accomplished through many different techniques ranging from cross-mediated breeding to advanced methods based on knowledge of genetics and genomics to the most advanced molecular technologies based on genome editing (GE). If MS sources have not been found in the species of interest or if their transfer to the productive varieties is hindered by technical limitations, MS can also be induced by mutagenesis with chemical or physical agents (Hawkes et al., 2011). At present, the ability to precisely recognize and edit DNA sequences can have a significant impact on functional genomics and crop advancement studies. The recent development of GE-based technologies has provided researchers with powerful tools not only for decoding gene functions but also for improving or introducing new plant traits. This progress offers an increasing number of approaches considered revolutionary in molecular biology since it allows modifications at genomic loci in a precise and efficient manner (Malzahn et al., 2017). GE methods, being less time-consuming, can accelerate the creation of new genetic variability with the accumulation of favorable alleles, able to dramatically change the biological process and resulting in a potential efficiency of cultivar development bypassing sexual crosses (Chen and Gao, 2014; Gao, 2015; Arora and Narula, 2017; Scheben et al., 2017). Furthermore, since the development of a new commercial male-sterile line using traditional breeding systems usually takes several years or decades, these modern genetic engineering techniques can reduce dramatically the breeding time (Zhou et al., 2016).

Starting from these assumptions, the aim of this manuscript is to provide a general overview of insights and advances into plant male sterility, first providing a brief description of conventional breeding programs, and then focus attention on the recent new breeding GE-based applications capable of inducing MS by targeting specific nuclear genic loci. In particular, a summary of the mechanisms underlying the recent CRISPR technology and relative success applications will be described for the main crop and ornamental species. Finally, we discuss the future challenges and potential opportunities of such technologies for introducing desirable alleles and improving many traits for precision breeding strategies.

## 2 MS based-conventional breeding methods

**Figure 1:**
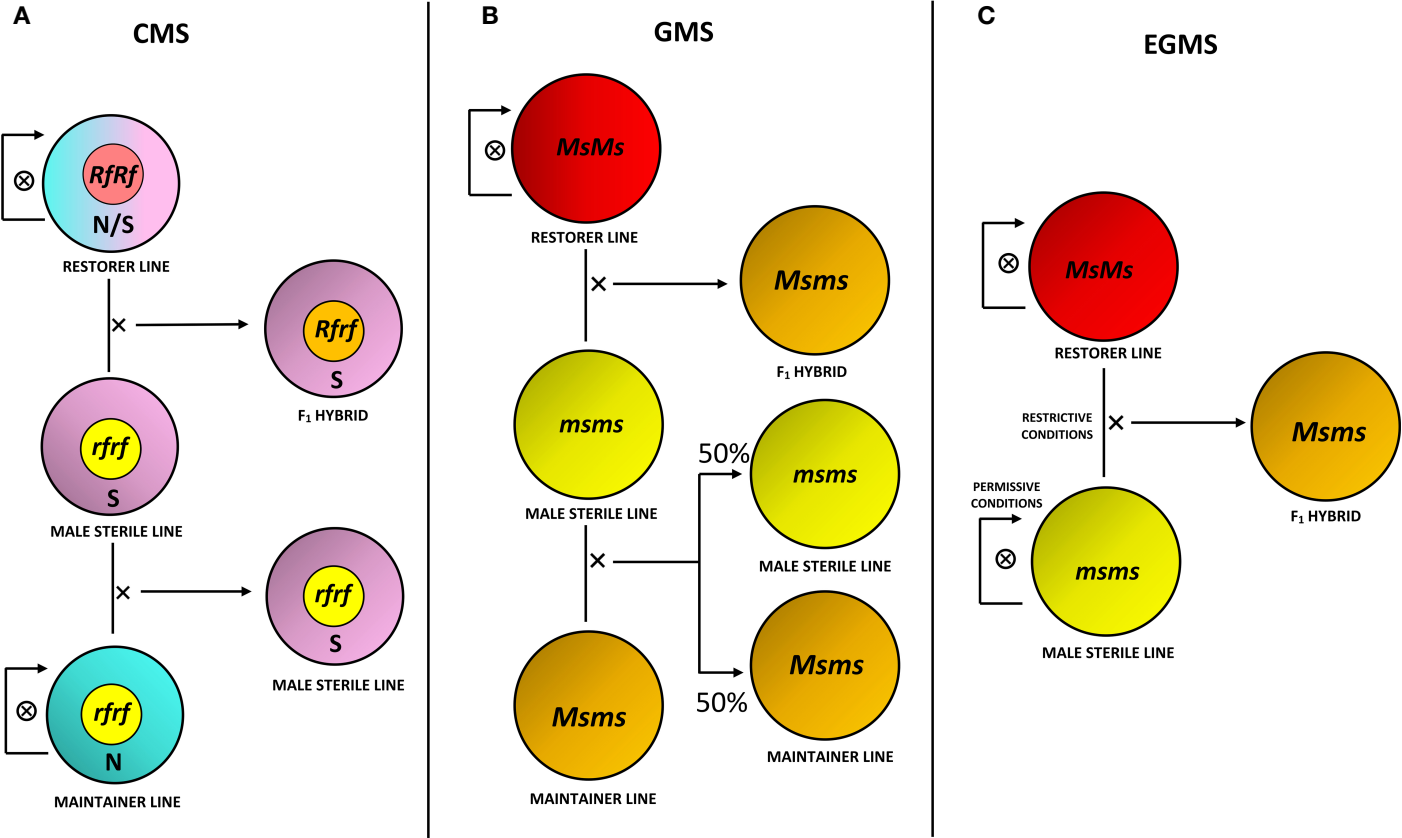
Production and maintenance strategies for MS systems. **(A)** Three-line system for *cytoplasmic male sterility* (CMS), involving an MS line with sterile cytoplasm (S) and restorer gene in recessive homozygous condition (*rfrf*), crossed with a maintainer line carrying normal fertile cytoplasm (N) and *rf* alleles for its maintenance, in addition to a fertility restorer line with N or S cytoplasm without distinction and restorer alleles in dominant homozygous, hence functional, condition (*RfRf)*, crossed with the MS line for F_1_ hybrid production. The F_1_ hybrid consequently brings S cytoplasm and is heterozygous for the restorer gene (*Rfrf*), hence male fertility. Maintainer and restorer lines are self-pollinated for their maintenance. **(B)** Three-line system for *genic male sterility* (GMS), involving a recessive homozygous MS line for the MS gene (*msms*), crossed with a heterozygous maintainer line (*Msms*) for maintenance, producing half recessive homozygous and half heterozygous progeny, in addition to a dominant homozygous restorer line (*MsMs*), crossed with the MS line for heterozygous and fertile F_1_ hybrid production. **(C)** Two-line system for *environmental genic male sterility* (EGMS), involving a recessive homozygous MS line, kept in permissive environmental conditions (i.e., low temperatures or short-day photoperiod) in order to make it become male fertile and self-pollinate for its maintenance, while kept in restrictive conditions (i.e., high temperatures or long-day photoperiod) to make it male sterile and to cross it with a dominant homozygous restorer line for F_1_ hybrid production.

The selection of improved varieties through conventional breeding primarily relies on phenotypic observations and the breeder’s experience. For planning a promising plant breeding program, the following association establishment between phenotype and relative genotype results is fundamental (Chen and Lubberstedt, 2010). As reported in the Introduction section, MS is an important trait for different purposes, primarily for the production of hybrid seeds. The development and propagation process of an F1 hybrid obtained through an MS system involves multiline maintenance strategies (Khan and Isshiki, 2016; Kim and Zhang, 2018; Xu et al., 2022; Scariolo et al., 2023). Various agronomic strategies can be used to introduce the MS trait in a commercial line of interest through conventional breeding approaches: after identifying naturally occurring male sterility within a species, the MS trait may be transferred to elite germplasm by cross pollination (Yamagishi and Bhat, 2014; Bruns, 2017; Zheng et al., 2020). Briefly, as schematically reported in **Figure 1**, CMS bases on a three-line system that includes, in addition to the MS line, a fertility restorer line and a maintainer line (Chen and Liu, 2014). Conversely, the use of the Mendelian recessive genes of GMS requires the discrimination of male fertile and sterile progeny prior to anthesis to ensure the maintenance of the MS line. This task can be challenging unless functional molecular markers associated with the MS locus are available (Wu et al., 2016). EGMS could overcome this problem by altering specific environmental conditions to make MS lines either male fertile or sterile (Sun et al., 2022).

The identification of functional molecular markers linked to specific traits can be of primary importance to allow future selection programs mediating marker-assisted selection (MAS), which identifies the following mapping gene responsible for the observed phenotype (Page and Grossniklaus, 2002; Schneeberger, 2014; Aklilu, 2021). The use of MAS results in fact in a useful predictive tool for the identification of male sterile genotypes, mapping markers closely associated with the MS locus (Mackenzie, 2012). The complexity and long times of these breeding strategies make the exploration of the molecular mechanisms a key feature to improve productivity and other traits of interest (Bohra et al., 2016; Van Ginkel and Ortiz, 2018; Yu et al., 2021a). On the basis of these articulated schemes of MS maintenance, defining the conditions in which the markers can be predictive molecular tools of genotype is a key point of each program. Furthermore, starting from the knowledge of a well-characterized mechanism of the MS system in model species, substantial genetic resources can be used for the discovery of homologous ms-related genes in other species (Leino et al., 2003; Fernandez Gomez and Wilson, 2014; Morales et al., 2022).

### 2.1 CMS system

Four different models have been described to explain how CMS can produce male sterility Chen and Liu (2014):

i) *Cytotoxicity model*: the proteins encoded by the CMS genes directly cause the death of the cells involved. At the basis of the mechanisms by which this occurs it has been hypothesized that there is mitochondrial dysfunction, but a well-defined model has not yet been developed, lacking molecular evidence of cytotoxicity itself. As a result, a simple explanation for CMS in these systems is that the CMS proteins cause mitochondrial malfunction in the anthers’ sporophytic or gametophytic cells, resulting in male abortion (Levings, 1993).

ii) *Lack of energy model*: the cellular respiration process is altered. In fact, CMS proteins can act as dysfunctional homologues of parts of complexes forming the electron transport chain, or changing proton gradients critical to the cellular respiration process, resulting in no ATP production. The molecular evidence supports the concept that some CMS are caused by an energy deficit in growing anthers, which demand more energy (Sabar et al., 2003; Wang et al., 2013).

iii) *Asynchronous programmed cell death* (PCD) *model*: PCD is induced in tapetum cells earlier than its normal course. It is implemented through the release of cytochrome C, a protein complex of the electron transport chain, and by increasing the production of reactive oxygen compounds (ROS, from Reacting Oxygen Species). By starting the autolysis before the pollen is mature, the tapetum cannot continue to nourish it and this does not complete its development. Plant male gametophytes form in anthers through cooperative contacts between sporophytic (anther wall) and gametophytic (microspore) cells, as well as correct PCD-controlled cellular degeneration of the tapetum, the deepest cell layer of the anther wall tissue (Mah, 2005).

iv) *Retrograde regulation model*: some CMS proteins are able to regulate the expression of nuclear genes, including some involved in the processes for correct reproduction. For example, they can disturb the formation activity of the stamens, in place of which carpels or petals develop. Or they can nullify the action of fertility restorative genes, when they are in their recessive allelic/haplotype form (Linke et al., 2003).

By comparing the proteomes of CMS and fertile lines, some CMS causative proteins, such as URF13 of maize CMS-T (Forde et al., 1978) and truncated COX2 of sugar beet CMS-G (Ducos et al., 2001), were discovered in other CMS systems. The CMS candidate genes were found in a few cases, such as radish CMS-Ogu (Bonhomme et al., 1991) and wheat alloplasmic CMS-AP (Rathburn and Hedgcoth, 1991), by analyzing the mitochondrial DNAs of segregating somatic hybrids (cybrids) produced from protoplast fusion between CMS-carrying lines and normal fertile lines. However, owing of the difficulties in acquiring cybrids and the uncertainty of recombination events between the mitochondrial DNAs of the fusion lines, this strategy is ineffective for most crops. Several methodologies can be used to identify CMS candidate genes. The most common approach is to look for changes in mitochondrial gene organization and/or mitochondrial transcriptome or proteome differences in CMS cytoplasm lines with and without the Rf gene(s). Nuclear Rf genes perform their action at different levels, involving various steps of protein synthesis or cellular metabolism (Chen and Liu, 2014).

Since in the case of CMS, 100% of offspring individuals will be MS, the use of molecular markers results a strategic key if identified and mapped in association with Rf genes. **Table 1** shows main examples of the important crop classes for which mapped and retrievable information regarding reproducible, codominant molecular markers linked to Rf genes, offering fast and reliable detection tools to select, by MAS, parental lines for production of the desired progeny (Jordan et al., 2010; Yan et al., 2017).

**Table 1:** Male sterility-related molecular markers mapped to Rf genes in CMS system.

	Species	Gene locus	CMS type	LG/Chrom	Molecular markers	Reference(s)
**Cereals**	**Maize**	Rf1	CMS-T (S)	3	RFLP	Schnable and Wise, 1994
		Rf2	CMS-T (S)	9	RFLP	Schnable and Wise, 1994
		Rf8, Rf*	CMS-T (S)	2L	RFLP	Dill et al., 1997
		Rf3	CMS-S (G)	2L	SSR, AFLP	Zabala et al., 1997; Zhang et al., 2006
		Rf4	CMS-C (S)	8	CAPS	Dewey et al., 1991; Liu et al., 2022
		Rf5	CMS-C(S)	5	RFLP	Sisco, 1991
	**Soybean**	Rf3	CMS (G)	9	CAPS, SSR	Sun et al., 2022
		Rf-m	CMS-M(G)	16	SSR	Wang et al., 2016
	**Wheat**	Rf1	CMS-T (S)	1A	SNP	Melonek et al., 2021
		Rf3	CMS-T (S)	1B	SNP	Geyer et al., 2016; Melonek et al., 2021
		Rf9	CMS-T (S)	6AS	SNP	Shahinnia et al., 2020
	**Rice**	Rf1	CMS-BT (G)	10	RFLP	Komori et al., 2004
		Rf4	CMS-WA (S)	10	SSR, SNP, InDel	Tang et al., 2014
		Rf2	CMS-LD (G)	2	CAPS, SNP	Itabashi et al., 2011
		Rf5	CMS-HL (G)	10	SSR	Hu et al., 2012
		Rf17	CMS-WA (S)	4	SNP	Fujii and Toriyama, 2009
		Rf98	CMS-RT98(G)	10	SSR	Igarashi et al., 2016
		Rf3	CMS-WA (S)	1	RAPD, RFLP, SSR	Zhang et al., 1997; Ahmadikhah and Karlov, 2006
		Rf6	CMS-BT (G)	8	SNP	Liu, 2004; Zhang et al., 2017; Zhang et al., 2019
**Horticultural**	**Pepper**	Rf	CMS-Peterson	6	SCAR, CAPS	Jo et al., 2016; Kang et al., 2022
**Non-food**	**Rapeseed**	Rf1	CMS-Pol (S)	18	RFLP	Jean et al., 1997
	**Cotton**	Rf2	CMS-D8(G)	19	RAPD, CAPS, AFLP, SSR	Wang et al., 2007
		Rf1	CMS-D2-2(S)	D5	SNP, InDel	Cheng et al., 2023; Wu et al., 2017
**Ornamental**	**Petunia**	Rf	NR	4	SSR	Bentolila et al., 1998
	**Sunflower**	Rf1	CMS-PET1(S)	13	SSR, TRAP	Yue et al., 2010
		Rf3	CMS-PET1(S)	7	SSR	Liu et al., 2012
		Rf5	CMS-PET1(S)	13	SSR	Qi et al., 2012
		Rf7	CMS-PET1(S)	13	SSR, SNP	Talukder et al., 2019
		Rf4	CMS-GIG2	3	SSR	Feng and Jan, 2008;
		Rf6	CMS-514A	4	SSR	Liu et al., 2013
		Msc1	CMS-PET1(S)	12	RFLP	Gentzbittel et al., 1999

The class to which each species taken into account belongs is reported alongside. The linkage group (LG) or chromosome (chrom), and available molecular markers for MAS application are indicated. CMS-type: S, sporophytic; G, gametophytic, indicates where the CMS acts. NR, Not reported.

In addition to major cereal crops and species, a great interest in mapping CMS-related loci has also been reflected in ornamental plants such as sunflower and petunia (Bentolila et al., 1998; Gentzbittel et al., 1999; Feng and Jan, 2008; Yue et al., 2010; Liu et al., 2012; Qi et al., 2012; Liu et al., 2013; Talukder et al., 2019).

### 2.2 GMS system

Several molecular mechanisms underlie GMS in different species, in many of them genes coding transcription factors are capable of modifying the expression of genes involved in reproductive processes. The result is disturbance of gamete formation, due to failure of homologous chromosome separation in meiotic anaphase I and delayed of programmed cell death in tapetum (Jeong et al., 2014). In particular, several nuclear genes have been found responsible for MS, causing arrest of microspore development. Furthermore, as mentioned in Introduction section, the male sterility can also depend on environmental conditions, and in this case the GMS is define as EGMS. Temperature-sensitive genic male sterile (TGMS) and photoperiod-sensitive genic male sterile (PGMS) lines were developed especially in cereals crops like rice and wheat. TGMS lines are sterile at high temperatures and fertile at low temperatures, while PGMS lines can either be sterile when the day is longer than the night and fertile when it is shorter, or vice versa. In China, EGMS lines occupy 20% of the area dedicated to the cultivation of hybrid rice (Li et al., 2007). Also in this case, the molecular markers result strategic keys if identified and mapped in association with the ms locus, as testified in past (Barcaccia et al., 2016). However, to date, in more than 610 species of flowering plants the MS trait is under investigation, and specifically in the past few decades, at least 40 GMS genes have been identified by MAS and characterized in model Arabidopsis and rice (Chen and Liu, 2014; Singh et al., 2019; Wan et al., 2019). As similarly reported in **Tables 1**, **2** shows main examples of the several crop classes for which mapped and retrievable information regarding reproducible, codominant molecular markers linked to nuclear male sterility genes. Molecular markers such as SSR, RFLP, SCAR, and SNP were fully employed for mapping male sterility genes, while insertion−deletion (InDel), target region amplification polymorphism (TRAP), sequence-related amplified polymorphism (SRAP), high resolution melting (HRM), and conserved orthologous set (COS) markers were sporadically used among these research studies. However, the data availability of mapped genes was correlated with species of great agronomic and economic importance: studies on cereals and other horticulture crop were prevalent, resulting in numerous mapped markers and associated genes involved in both MS systems, which offer open access for hybrid production using male-sterile lines (Li et al., 2007; Rout et al., 2021; Morales et al., 2022). These insights were interesting because proper MAS application could offer competitive phenotypes for market demand and contribute to reducing production costs, which is also fundamental for ornamental plant companies.

**Table 2:** Male sterility-related molecular markers mapped to relative genes associated with the GMS trait.

	Species	Gene locus	Type	Gene function	Role in ms	LG/Chrom	Molecular markers	Reference(s)
**Cereals**	**Maize**	Ms30	GMS	GDSL Lipase	pollen exine formation, anther cuticle development	4	SNP	An et al., 2019
		ms39	GMS	callose synthase12 (ZmCals12)	pollen development, plant height, tassel length, tassel branch number	3	InDel, SSR, SNP	Zhu et al., 2018; Niu et al., 2023
		Ms28	GMS	ZmAGO5c protein	regulation of the tapetum development	5	InDel, SSR, SNP	Li et al., 2021
		ms40	GMS	bHLH transcription factor	tapetum degeneration retardation	4	InDel	Liu et al., 2021
		ms32	GMS	bHLH transcription factor	regulator of both division and differentiation during anther development	2	RFLP	Moon et al., 2013; Chaubal et al., 2000
		Ms33	GMS	glycerol-3-phosphate acyltransferase (GPAT)	tapetum development and metabolism disruption	2L	SSR	Xie et al., 2018
		Ms7	GMS	PHD finger transcription factor	abnormal microspore wall and tapetal cell development	7	SSR	Zhang et al., 2017
		Ms20	GMS	irregular pollen exine1 (ipe1)	anther cuticle and pollen exine formation	1	SSR	Wang et al., 2019
	**Soybean**	mst-M	GMS	NA	NA	13	CAPS, SSR	Zhao et al., 2019
		ms1	GMS	kinesin protein	cell plate formation in male gametogenesis	13	SSR	Yang et al., 2014b; Fang et al., 2021
		ms6	GMS	R2R3 MYB (GmTDF1-1) transcription factor	anther development regulator	13	SSR	Yang et al., 2014b; Yu et al., 2021
		ms4	GMS	PHD protein	failure of cytokinesis after telophase II, coenocytic microspores	2	SSR	Yang et al., 2014b; Thu et al., 2019
	**Wheat**	ms5	GMS	GPI-anchored nsLTP	pollen development	3AL	SNP	Pallotta et al., 2019
		ms1	GMS	GPI-anchored nsLTP	pollen exine development	4BS	SSR, CAPS, SNP	Yang et al., 2021; Tucker et al., 2017; Wang et al., 2017
		Ms3	GMS	NA	NA	5A	centromere-related	Maan et al., 1987
	**Rice**	rpms1	rPGMS	NA	NA	8	SSR	Peng et al., 2008
		rpms2	rPGMS	NA	NA	9	SSR	Peng et al., 2008
		tms12-1	P/TGMS	small RNA osa-smR5864w	regulator of the development of the male reproductive organ	12	SSR	Zhou et al., 2012
		tms2	TGMS	alpha-galactosidase precursor (ORMDL)	sphingolipid homeostasis, pollen development	7	SSR	Lopez et al., 2003; Chueasiri et al., 2014
		tms3(t)	TGMS	NA	NA	6	RAPD, RFLP	Subudhi et al., 1997
		pms2	PGMS	NA	NA	3	RFLP	Zhang et al., 1994
		pms4	PGMS	NA	NA	4	SSR	Huang et al., 2008
		pms1	PGMS	21-PHAS gene	differential accumulation of the phasiRNAs	7	SSR, CAPS,InDel	Fan et al., 2016
		tms5	TGMS	RNase Z-S1	defective pollen production	2	CAPS, AFLP	Wang et al., 2003; Yang et al., 2007; Zhou et al., 2014
		tms4(t)	TGMS	NA	NA	2	AFLP, RFLP, SSR	Dong et al., 2000
		rtms1	rTGMS	NA	NA	10	AFLP	Jia et al., 2001
		tms6	TGMS	NA	NA	5	STS, SSR	Lee et al., 2005
		pms3	PGMS	long noncoding RNAs (lncRNA)	pollen development of plants grown under long-day conditions	12	RFLP	Mei et al., 1999; Ding et al., 2012
**Horticultural**	**Chicory**	ms	GMS	MADS-box gene	flower development	4	SSR	Cadalen et al., 2010
		ms-myb80	GMS	Myb 80 transcription factor	anther development	9	SSR, CAPS, SNP	Barcaccia and Tiozzo, 2012; Palumbo et al., 2019
		NMS	GMS	hypothetical S-domain RLK gene	anther development	5	SSR, SCAR	Cadalen et al., 2010; Gonthier et al., 2013
	**Pepper**	ms1	GMS	PHD finger transcription factor	sporophytic factor controlling anther and pollen	5	HRM	Jeong et al., 2018
		ms3, msw	GMS	NA	NA	1, 5	CAPS	Naresh et al., 2018
		ms8	GMS	NA	abortion of microspore formation	P4	SCAR	Bartoszewski et al., 2012
		ms10	GMS	NA	NA	1	SSR	Aulakh et al., 2016
	**Tomato**	ps2	GMS	polygalacturonase gene (PG)	blocking anther dehiscence, fruit ripening	4	COS	Gorguet et al., 2006; Gorguet et al., 2009
		ms10	GMS	anthocyanin-related GST gene (SlGSTAA)	role in anthocyanin transport	2	InDel	Zhang et al., 2016
		ms32	GMS	bHLH transcription factor	pollen and tapetum development	1	InDel	Liu et al., 2019
		ms15²^6^	GMS	B-class MADS-box TM6	stamen development	2	InDel	Cao et al., 2019
**Non-food**	**Rapeseed**	ftms	GMS	putative β-(1,3)-galactosyltransferase (Bra010198)	microspore development	A05	SSR	Tan et al., 2019
		ms3	GMS	Tic40 protein	tapetal function and pollen development	N19	SCAR	Huang et al., 2007; Zhou et al., 2012
		Ms-cd1	GMS	*SALT-INDUCED AND EIN3/EIL1-DEPENDENT 1 (SIED1)*	primary pollen mother cell (PMC) and microspore formation	3	SRAP	Zhang et al., 2011; Liang et al., 2017
	**Cotton**	ms5, ms15	GMS	NA	NA	12	SSR	Chen et al., 2009
		ms6	GMS	NA	NA	26	SSR	Chen et al., 2009
**Ornamental**	**Marigold**	Tems	GMS	B class MADS-box genes	floral organ homeotic conversion of the petals and stamens	NR	AFLP, SCAR	He et al., 2010

The class to which each species taken into account belongs is reported alongside. The linkage group (LG) or chromosome (chrom), and available molecular markers for MAS application are indicated. NA, Not Applicable. rPGMS, reverse photoperiod-sensitive genic male sterility. rTGMS, reverse temperature-sensitive genic male sterility. P/TGMS, photoperiod, temperature-sensitive male sterility.

## 3 A new frontier of precision plant breeding technology: GE mediated by the CRISPR/Cas system

**Figure 2:**
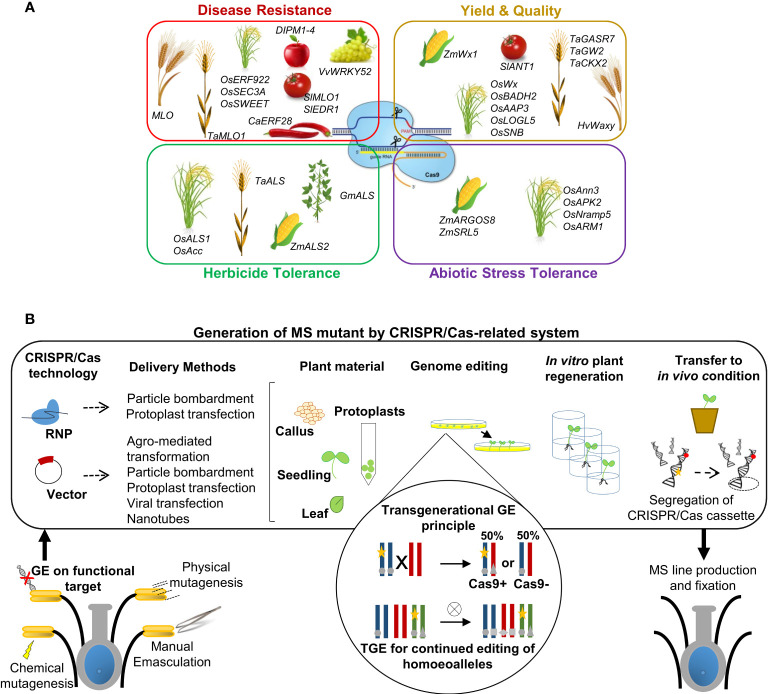
GE applications in precision plant breeding approaches. **(A)** Schematic representation of the main applications of GE for crop improvement through CRISPR/Cas and related systems. Examples of genes modified for improving specific traits are listed in each specific box for various reference crops. **(B)** Simplified representation of the workflow for MS generation mediated by CRISPR/Cas technology applied to target genes. In summary, gene editing is induced by transient or stable expression of a Cas nuclease and gRNA through the transformation/transfection of the ribonucleoprotein (RNP) complex or DNA vector. Both CRISPR machinery reagents can be delivered into plant cells using biolistic transformation or other methods, according to suggested transformation/transfection protocols related to species, plant tissues and the methodological approach followed. Such events can lead to the generation of edited whole plants. The transgene locus is usually heterozygous in the first generation of transgenic plants (T0). Afterwards, elimination of the CRISPR/Cas cassette transgene (yellow star) by genetic segregation, according to Mendelian genetics, occurred to obtain transgene-free material. Transgene-free and edited T1 plants can be identified by PCR-based genotyping. The transgenerational GE principle and potential applications in plants are highlighted schematically in circles: transgenic plants represented as a chromosome pair are hemizygous for a CRISPR/Cas9-containing T-DNA locus (yellow stars) and edited in both alleles (grey circles). When crossed with a WT, the resulting progeny either lacks the T-DNA and inherits a single edited allele or inherits the T-DNA, resulting in (transgenerational) editing of the inherited WT allele. TGE for continued editing of homoeoalleles in polyploids species: a transgenic line may have edits only in a subset of homoeoalleles at the homologous chromosomes. After self-crossing and selecting plants that inherited the T-DNA, all homoeoalleles may now be edited. The details can be found within the text.

Novel GE technologies have been intensively developed through diverse biological systems depending on sequence-specific nucleases (SSNs). Upon induction, all SSNs may detect a specific DNA fragment and cause double-stranded breaks (DSBs), repaired by two endogenous repair machinery of plant. Initially, ZFNs (zinc-finger nucleases) and TALENs (transcription activator-like effector nucleases) were the two systems primarily employed in genome editing techniques (Smith et al., 2006; Petolino, 2015; Malzahn et al., 2017). However, the difficulties of array and vector design in each of these methods, as well as the time-consuming work necessary to construct vectors for each new DNA sequence target, have hampered their widespread usage for plant genome editing. In contrast, with subsequent scientific breakthroughs, CRISPR/Cas-based genome editing systems (clustered regularly interspaced short palindromic repeats/CRISPR-associated protein) have been increasingly employed in the last decade (Li et al., 2013; Nekrasov et al., 2013; Shan et al., 2015). Their use is constantly expanding in numerous applications, resulting in a wider array of editing tools developed for several purposes (**Figure 2**). CRISPR/Cas-based systems are considered more robust and simpler for targeting gene editing since they present a significant advancement over previous systems, such as the simplicity and versatility in vector design and construction for subsequent plant transformation (Chen et al., 2019; Bhat et al., 2020; Nadakuduti and Enciso-Rodriguez, 2020; Zhu et al., 2020). An increasing number of studies attest to the expanded applications of Cas9 nuclease for editing beyond double strand breaks, and the accompanying benefits of those systems have resulted in quick, widespread acceptance for editing applications in a diverse range of plant species. Cas9-related nuclease, if associated with an RNA guide (single guide RNA, sgRNA), is able to identify a special site PAM (Protospacer Adjacent Motif) in the host DNA and cut the target sequence recognized, mediated by the complement to which the sgRNA binds (Mojica et al., 2009; Jinek et al., 2012), inducing the development of modified and improved forms of Cas9 and Cas9-like nucleases. In addition to Cas9, other related enzymes (Cas12a, CasΦ, and Cms1), derived from other CRISPR systems, have been implemented since they are potentially useful for editing approaches, each with slightly different capabilities to recognize and modify PAM sites (Zetsche et al., 2015; Begemann et al., 2017; Gao et al., 2017; Li et al., 2018; Pausch et al., 2020).

This approach is defined as a precision-type plant breeding technology, and it is currently being utilized to change the characteristics of various plants, including important crops, as well as to produce new germplasm resources. (Gaillochet et al., 2021). The applications have been primarily focused on traits related to stress tolerance, disease resistance, quality improvement, and higher yields with minimal input (Liu et al., 2022) (**Figure 2A**). In particular, the CRISPR/Cas system has been widely employed to edit plant genomes to modify genes in various ways, e.g., gene knockout, gene knock-in, gene regulation, base editing, and prime editing (Zhang et al., 2021a). Gene knockout/-in and base and/or prime editing mediated by CRISPR/Cas-related tools have resulted in flexible and successful strategic ideas used to alter the function of key genes and their associated networks, regulating complicated crop traits (Gaillochet et al., 2021). The successes of CRISPR/Cas application in crop improvement have been reviewed in many papers, which are focused on the improvement of crop resistance to fungi, bacteria, and viruses, typically mediating targeting susceptibility systems to increase resistance (Wang et al., 2014; Malnoy et al., 2016; Wang et al., 2016; Nekrasov et al., 2017; Borrelli et al., 2018; Ma et al., 2018; Pu et al., 2018; Wang et al., 2018b; Dong and Ronald, 2019; Oliva et al., 2019; Mishra et al., 2021), resistance to an assortment of herbicides (Li et al., 2015; Chen et al., 2017; Zong et al., 2018; Zhang et al., 2019; Kuang et al., 2020), and abiotic stresses such as drought, salinity, high temperatures, and soil pollution (Lou et al., 2017; Nieves-Cordones et al., 2017; Shi et al., 2017; Tang et al., 2017; Pan et al., 2020). In particular, the main purposes of food crop improvement research using GE technology are to raise yield (e.g., grain size/weight/number per panicle) and crop quality traits determined by internal (e.g., contents of nutrients and bioactive substances) and external (e.g., size, color, and texture) factors related to a response to adverse surrounding environments (Shan et al., 2015; Zhang et al., 2016b; Lou et al., 2017; Dahan-Meir et al., 2018; Wang et al., 2018c; Chen et al., 2019; Ma et al., 2019; Voss-Fels et al., 2019; Wang et al., 2020; Zhu et al., 2020; Liu et al., 2021b). To simplify the overview of this complicated scenario, **Figure 2A** summarizes the main traits that can be enhanced by genome editing, with a list of example genes edited by the CRISPR/Cas system to improve related plant species.

## 4 CRISPR/Cas system applications for producing MS

In addition to stress response, and traits related to quality and yield, CRISPR/Cas-based technology offers a new strategic tool to affect other crop traits associated to fertility/sterility (**Figure 2B**). As described in section 2, researchers have employed several strategies for integrating MS traits into genomes of interest using information and methods arising from conventional breeding approaches, with the aim to guarantee high varietal purity breeding and to have better offspring in terms of uniformity, yield, and stress tolerance (Bao et al., 2022). Thanks to important forward genetic tools, as mutagenesis approaches and TILLING populations, it has been possible to discover and investigate new candidate genes controlling male sterility. Furthermore, the increasing number of transcriptomic and proteomic studies in recent decades, mostly on crop species, has allowed us to characterize an emergent number of genes with different roles in the development of male reproductive organs and consequently with a putative role in MS induction. If the role of these genes is confirmed as influencer of MS trait, they could be potential targets for subsequent gene editing strategies (Carroll, 2011; Li et al., 2012). The elucidation of molecular processes regulating anther and pollen development has increased the identification and characterization of new putative candidate male-sterility genes (MSGs) in several species, allowing the development and effective use of numerous biotechnology-based male-sterility systems for crop hybrid breeding (Perez-Prat and Van Lookeren Campagne, 2002; Whitford et al., 2013; Wu et al., 2016; Zhang et al., 2019). As reported in more detail in following sections, CRISPR/Cas technology is resulted a novel, rapid and alternative method for the generation of MS lines through target gene editing, both in food crops (monocots and horticultural dicots) and in the increasing ornamental sector, implementing also the knowhow underlying male sterility in plants (Cong et al., 2013; Wang et al., 2018a).

### 4.1 Generation of MS mutant by CRISPR/Cas-related system

The CRISPR/Cas system, especially based on Cas9, has been successfully applied for the generation of male sterile lines in important worldwide food crops (Barman et al., 2019; Okada et al., 2019). Studies in main crops, such as rice, soybean, maize, and tomato, have reported that pooled CRISPR/Cas9 methods can result in valid strategies to generate a population of mutants for the MS trait (Jacobs et al., 2017; Meng et al., 2017; Liu et al., 2019; Liu et al., 2020). The application of CRISPR/Cas technologies for generating mutants with a male sterile phenotype is an effective tool, mainly mediating a knock-out approach towards target GMS genes with nuclear origin since, compared to cytoplasmic male sterile lines, it is much easier and more useful to produce hybrid seeds (Qi et al., 2020).

Generally, MS mutants result from mutations in target genes involved in microsporogenesis and/or microgametogenesis. Meiosis-related, tapetum-specific and transcription regulatory genes, such as eme1/exs, tpd1, ams and ms1, have been elucidated as key candidate genes involved in these biological processes (Canales et al., 2002; Zhao et al., 2002; Yang et al., 2003). Furthermore, many Arabidopsis transcription factors (TFs) genes, such as MYB103, DYT1, TDF1, AMS, bHLH10, bHLH89 and bHLH91, have been investigated as direct controllers of pollen development (Sorensen et al., 2003; Zhang et al., 2006; Zhang et al., 2007; Zhu et al., 2008; Zhu et al., 2015; Pan et al., 2020). The molecular and functional information has been then easily transferred from models to crops, as reported for Arabidopsis and rice, in which two analogous pathways regulating pollen and tapetum development have been identified in previous research (Fu et al., 2014; Jeong et al., 2014; Zhu et al., 2015; Mishra et al., 2018). In tomato, two homologous genes have been identified as regulators of tapetum and pollen formation. The first, *SlMS10* (Solyc02g079810_*ms10^35^*) gene, encoding a basic helix-loop-helix TF (bHLH) and homologue to *AtDYT1* and *OsUDT1*, carries both PCD and meiosis alteration in the tapetum during microsporogenesis (Jeong et al., 2014). Its editing has confirmed that *SlMS10* is a possible good target candidate for male sterility induction since its knockout mediated by the CRISPR/Cas9 system conferred a male sterility phenotype (Jung et al., 2020). Recently, Liu and colleagues demonstrated that the creation of a mutation in *ms10^35^* by CRISPR/Cas9 technology in association with its linkage marker genes led to marker use for creating mutants exhibiting complete male sterility and recognition during the early developmental stage, confirming promising application possibilities in the production of hybrid seeds (Liu et al., 2021a). The second, Solyc01g081100, homologous to the AtbHLH10/89/90 and *OsEAT1* genes, is a candidate gene for the *male sterile 32* (*ms32)* mutant, a locus affecting tapetum and pollen development, and for this reason, it is suggested as a good target for gene editing to quickly develop such lines of interest (Liu et al., 2019). Furthermore, knockout by CRISPR/Cas9 of the *SlAMS* gene, encoding another basic helix-loop-helix (bHLH) TF, caused downregulation leading to abnormal pollen development, which in turn decreased pollen viability and subsequently generated a male-sterile phenotype (Bao et al., 2022). Recently, other tomato CRISPR/Cas9-edited lines with male sterility phenotypes were obtained by knock-out of *SlPHD*_*ms1* (Solyc04g008420), encoding a PHD-type TF involved in pollen formation and tapetum development, suggesting a key role for SlPHD in male sterility and aiding research into the regulatory processes of pollen and tapetum growth in tomato (Gökdemir et al., 2022). With analogous purposes, CRISPR/Cas technology was also applied in other horticultural crops, as demonstrated in cucurbit species. For example, the knockout of *eIF4E* by CRISPR/Cas9 in melon highlighted for the first time the association between *eIF4E* editing and the development of male sterility (Pechar et al., 2022). In watermelon (*Citrullus lanatus* L.), knockout of *ClATM1* by CRISPR/Cas9 causes male sterility, confirming its self-regulatory activity and providing new insights into the molecular mechanism underlying anther development (Zhang et al., 2021b).

In monocots, several CRISPR/Cas systems for producing MS have been reported as successful applications in precision breeding. An improved CRISPR/Cas9 system was driven by the TaU3 RNA polymerase III U3 promoter, and three homologous alleles expressing the wheat redox enzyme NO POLLEN 1 (NP1) were altered to produce totally male-sterile wheat mutants (Li et al., 2020). Furthermore, with recent molecular identification of the *Ms1* gene and exploiting strategies related to transgenerational gene editing (see below section 5.2), it has been possible to extend the use of the CRISPR/Cas9 system to generate *Ms1* knockout wheat lines that exhibit male sterility in the first generation, demonstrating the utility of the CRISPR/Cas9 system for the rapid generation of nuclear male sterility in hexaploid species like wheat (Okada et al., 2019). Chen and colleagues created a CRISPR/Cas9 vector in maize to target the male sterility gene 8 (Ms8). The resulting mutant was male-sterile, which was compatible with Mendelian genetic rules and was stably acquired by subsequent generations (Chen et al., 2018b). Furthermore, editing *ZmMTL* (ZmPLA1) with the CRISPR/Cas9 system has produced maternal haploid inducers with powerful haploid identification markers useful for breeding doubled-haploid crops, such as maize itself (Dong et al., 2018). Additionally, *ZmMS26*, a known nuclear fertility gene (Loukides et al., 1995; Djukanovic et al., 2013) that is conserved in other monocots, like rice, wheat, and sorghum (Cigan et al., 2017), was subjected to precision editing: targeted mutagenesis of MS26 utilizing the modified I-CreI homing endonuclease or CRISPR/Cas9 resulted in the generation of new ms26 male sterile lines (Djukanovic et al., 2013; Svitashev et al., 2015; Qi et al., 2020). In rice, gene knockout by CRISPR/Cas9 of the *OsHXK5* gene resulted in male sterility, contributing to demonstration that OsHXK5 contributes to a large portion of the hexokinase activity necessary for the starch utilization pathway during pollen germination and tube growth, as well as for starch biosynthesis during pollen maturation (Lee et al., 2020).

### 4.2 Environmental genic male sterility

The success of CRISPR/Cas technology application has also been provided in EGMS conditions, or rather the ability to switch from fertile to sterile conditions and vice versa, by adjusting environmental variables such as temperature and photoperiod. Great progress has been recently achieved in the understanding of PGMS or TGMS traits in cereal crops, and several genes controlling P/TGMS traits have been investigated and transferred, mediating conventional breeding and/or biotechnological transformation, in specific lines on which more than 30% of cereal hybrid production depends in China (Ding et al., 2012; Zhou et al., 2012; Zhang et al., 2013; Huang et al., 2014; Zhou et al., 2014). Several studies elucidated the molecular genetic mechanisms at the base of EMGS, confirming also the interesting role assumed by phasiRNAs (phased small-interfering RNAs) generated by long-noncoding RNAs. In rice, for example, the phasiRNAs originated from PMS1T locus regulates PSMS in rice (Fan et al., 2016). Especially in rice, in the last few years, many genes influencing PGMS or TGMS traits have been discovered and cloned, and several reports describe different CRISPR/Cas-based approaches to obtain photo- and thermosensitive male-sterile lines. For example, a simple and efficient rice TGMS cultivation system using CRISPR/Cas9 editing technology was proposed to knock out the *TMS5* (*thermosensitive genic male-sterile 5*) gene target, with great value in new commercial “transgene free” TGMS rice lines (Zhou et al., 2016). *TMS5* is a nuclear recessive gene that controls the TGMS trait and extensively used in two-line hybrid rice breeding. It was the first spontaneously mutated *Oryza sativa* ssp. indica, identified more than 30 years ago, and encodes an RNase ZS1 endonuclease, able to degrade the temperature-sensitive ubiquitin fusion ribosomal protein L40 (UbL40) mRNA (Zhou et al., 2014). A study found that when plants were grown under a high temperature regime, several tms5 mutants developed in a background of the japonica type showed a high degree (85.3%) of pollen sterility (Zhou et al., 2016), confirming that targeted modification of TMS5 by the CRISPR/Cas9 system is a successful approach to develop TGMS lines for hybrid rice production. Huang et al. targeted the TMS5 gene, producing a mutant that was entirely male-sterile at high temperatures but male-fertile at low temperatures, with a pollen fertility transition temperature fixed at 28°C. (Huang et al., 2014). Recent studies revealed the molecular mechanism of *tms5* leading to male sterility in rice to easily obtain excellent TGMS lines (Fang et al., 2022) and potentially applicable in other crops. CRISPR/Cas9-engineered mutation of *TMS5* also resulted in the formation of thermosensitive male sterility in maize (Li et al., 2017). In addition, Li et al. altered the carbon starvation *CSA* gene in pollen grains of the rice variety ‘Kongyu 131’ and found that the *csa* mutant had a male-sterile phenotype in short-day and a male-fertile phenotype in long-day conditions. (i.e., photosensitive nuclear male sterile mutant) (Li et al., 2016), whereas in tomato, Shen and colleagues generated photosensitive/thermosensitive male-sterile lines by using CRISPR/Cas9 modifying the genic male-sterile 2-2 (PTGMS2-2) gene (Liu et al., 2019).

## 5 New potential applications of CRISPR/Cas systems in MS mutant production

### 5.1 Generating CRISPR-edited DNA-free by transient transformation system

As described in the previous sections, site-specific genome editing by CRISPR/Cas9 technology is becoming a progressively more successful tool for functional, basic and applied plant research because it can generate a high rate of mutation while being relatively easy to use (Zhang et al., 2013; Lowder et al., 2015; Ma et al., 2015; Wang et al., 2016). Numerous methods have been used to create CRISPR-edited plants devoid of CRISPR constructs and other transgenes because the lack of any transgenes in gene-edited plants is a requirement for the commercialization of any CRISPR-edited plants with stable valuable traits. For public approval, gene elimination or bypassing alien elements to edit endogenous genes is fundamental and could be a strategic approach, even if transgenic intermediates are transiently necessary (**Figure 2B**). The main different strategies useful to avoid the maintenance of transgene integration have been deeply described by He and Zhao, 2020 (He and Zhao, 2020). Commonly, after CRISPR-mediated mutagenesis, the Cas9 gene and associated DNA sequences are eliminated through genetic segregation, which frequently allays public concerns about genetically modified individuals. The biggest advantage of the method is that it could allow the selection of plants that no longer contain the T-DNA sequence, producing plant materials not containing any foreign DNA even though they were produced using transgenic technology mediating stable transformation methodologies. However, the fact that many commercial crop varieties are polyploid, heterozygous, or asexually reproduced complicates these efforts. Many commercial cultivars’ genome complexity, long juvenile phase, and/or self-incompatibility limit the development of CRISPR-mediated transgenic crops since backcrossing is required to remove the CRISPR transgene.

In the past, plant transient transformation technology has been widely used as an alternative approach to facilitate rapid and efficient gene function analysis (Sheen, 2001; Chen et al., 2006). Using transient transformation methods, such as particle bombardment (Romano et al., 2003), transient transformation by *Agrobacterium* sp. (Cui et al., 2017) and polyethylene glycol (PEG)-mediated protoplast transfection (Cankar et al., 2022), excellent results in plant research have been achieved. Among these, the protoplast transient expression system has played a relevant role in genomics and proteomics research, resulting in a potential, rapid, and convenient technique for testing new technologies, such as GE approaches. In general, transient expression methods for protoplasts have been designed for many crop species, including monocots, dicots, herbaceous and woody species, such as rice (Yang et al., 2014a), barley (Bai et al., 2014), corn (Cao et al., 2014), apple (Maddumage et al., 2002), and grapevine (Zhao et al., 2016). These findings demonstrate the possibility and feasibility of utilizing protoplasts for CRISPR-mediated gene editing, particularly in species with a protracted juvenile phase, heterozygosity, or asexual propagation. Likewise, this strategy could represent the most feasible way to directly apply CRISPR-mediated DNA-free genome editing technologies for improving traits and increasing commercial value, as already experimentally confirmed for food and non-food crops, such as strawberry (Martin-Pizarro et al., 2019; Wilson et al., 2019), potato (Gonzalez et al., 2019; Nicolia et al., 2021; Zhao et al., 2021), lettuce (Woo et al., 2015), chicory (De Bruyn et al., 2020; Cankar et al., 2022), *Nicotiana tabacum* (Lin et al., 2018; Hsu et al., 2019) and *Brassica oleracea* (Lee et al., 2020; Hsu et al., 2021), and ornamental species, as petunia (Yu et al., 2021b). For these reasons, protoplast transient expression systems represent a promising and valid approach for generating CRISPR-edited DNA-free plant material and MS mutant production (**Figure 2B**). Numerous studies describe the different gene modification methods using transient expression of the Cas protein and associated sgRNA, mediating the main delivery methods into somatic plant cells, which may be done either as DNA vectors, through Agrobacterium infiltration (Chen et al., 2018a), or as ribonucleoprotein (RNP), using biolistic delivery (Liang et al., 2018), nanotubes (Demirer et al., 2019), virus transfection (Ellison et al., 2020), PEG-calcium (PEG–Ca^2+^) (Toda et al., 2019). Because there is no foreign DNA present during transfection, direct transfection of the RNP complex eliminates the risk of plasmid DNA insertions into the plant genome (Andersson et al., 2018). Genome editing is realizable utilizing protoplasts without the insertion of foreign CRISPR DNA and without the necessity for hybridization, introgression, or back-crossing of progeny in the T0 generation. Furthermore, protoplasts are single cells that are edited before the first cell division: new plants grow from a single modified protoplast, ensuring that all cells share the same genetic background and that edited alleles are passed down to the next generation However, RNP-mediated genome editing has been employed successfully in many plant species, targeting genes with agronomic interest, involved disease resistance (Malnoy et al., 2016), in grain yield (Toda et al., 2019), nutritional composition (Andersson et al., 2018), and male fertility (Svitashev et al., 2016). MS induction, using an analogous approach, has been successfully achieved only in maize. Svitashev and colleagues demonstrated the success of their research, in which two male fertility nuclear genes (*MS26* and *MS45*) were targeted by purified Cas9 protein preassembled with *in vitro* transcribed gRNAs, demonstrating DNA-free genome editing in a major crop species using biolistically delivered Cas9–gRNA RNPs on immature embryos and subsequent plant regeneration (Svitashev et al., 2016). These positive results suggest the potential of applying similar methodologies in other large crops to increase the number of examples of male sterile lines CRISPR-edited DNA-free by transient transformation system by RNP complex.

### 5.2 Transgenerational gene editing

Because CRISPR/Cas9 expression cassettes and target sites are distributed throughout the genome, segregation and deletion of CRISPR/Cas9 cassettes is conceivable through subsequent selfing or crossing (**Figure 2B**). However, in crops with a high level of genome complexity, highly heterozygous, polyploid genomes, and usually propagated vegetatively, this is not easily achieved. Specifically, efficient propagation and stacking of first-generation mutations becomes increasingly difficult or nearly impossible with polyploidy.

Numerous new strategies have been developed to extend the CRISPR toolbox, and many of these new schemes could also take advantage from *transgenerational gene editing* (TGE)-based strategies, defined as the continued ability of Cas9 to edit also after cross: this means that if the Cas9 nuclease is still active, after cross it will encounter a new WT allele, which can be edited to create independent alleles. TGE has been utilized for a variety of applications, some of which are not always defined as TGE, such as the editing new alleles in polyploid crops, the creating allelic variation, and the editing target genes in refractory genetic backgrounds (Impens et al., 2022). Mutations are frequently found only in a fraction of the homoeoalleles targeted by the same sgRNA in polyploid crops such as hexaploid common wheat (*Triticum aestivum*) and tetraploid cotton (*Gossypium hirsutum*) (Wang et al., 2018a; Wang et al., 2018b; Wang et al., 2018c). While expressing CRISPR/Cas9 for more than one generation during TGE promotes on-target homoeoallele editing, it does not always boost off-targeting.

On the basis of TGE, with the purpose of accelerating the understanding of MS and ensuring speedy improvement, a new approach (Ramadan et al., 2021) was tested for example in cotton system, in which the use of pooled sgRNAs targeting single or duplicated genes belonging to different families provided a large number of intentional mutants that would help us know male sterility in cotton itself. Furthermore, this strategy ensured a rapid characterization of the key genes which may influence fertility in cotton, with important consequences for cotton future genetic improvement (Ramadan et al., 2021). Furthermore, as previously mentioned, a TGE-based methodology was implemented to facilitate the ongoing modification of homoeoalleles in species like hexaploid wheat, which is not easily amenable to conventional mutagenesis techniques. In this approach, a transgenic line may exhibit modifications in only a subset of homoeoalleles. However, through self-crossing and careful selection of plants inheriting the T-DNA, it becomes possible to modify all homoeoalleles. Singh and colleagues proposed an effective utilization of the CRISPR/Cas system and next-generation sequencing for mutant analysis in wheat. They successfully established the role of TaMs26 in wheat pollen generation by combining mutations in TaMs26 from the A-, B-, and D-genomes through crossing, resulting in the development of male sterile plants (Singh et al., 2017). Orthologous Ms26 mutations in rice and sorghum plants, as in maize, confer a recessive male sterile phenotype, and restoration of fertility in these mutant sorghum plants was achieved by a copy of maize Ms26 (Cigan et al., 2017). Afterwards, with recent molecular identification of the male fertility Ms1 gene, it has been possible to extend the use of the CRISPR/Cas9 system to generate Ms1 knockout wheat lines with male sterility in the first generation, demonstrating the potential of the CRISPR/Cas9 system for the fast generation of GMS in hexaploid wheat (Singh et al., 2018; Okada et al., 2019).

This evidence on transgenerational gene editing activity demonstrates that TGE can contribute to novel variation in the offspring of CRISPR/Cas9-expressing plants, and that Cas9-inducible trait can be transferred by crossing the plants expressing the gene editing constructs with the lines of interest.

## 6 Potential application of the CRISPR/Cas system in MS ornamental species: open perspectives

The interest in obtaining MS lines by molecular precision breeding mediated by the CRISPR/Cas system has been generally described as a fundamental step for the production of F_1_ hybrids in horticultural crops. In contrast, in ornamental plant research, this aspect has not been deeply investigated to date, despite an increasing number of studies on potential CRISPR/Cas system applications in precision breeding in ornamental plants being continually tested for improving several traits. In fact, in ornamental species, where traits such as high heterozygosity, large genomes, high chromosome numbers, polyploidy, long life cycles, self-sterility, or the inability to produce seeds frequently limit the applicability of conventional breeding methods, genome editing approaches are particularly desirable (Azadi et al., 2016; Sharma and Messar, 2017). Furthermore, obtaining nontransgenic first-generation altered plants and permitting the development of foreign DNA-free editing approaches would be extremely beneficial in such instances. However, the potential of using such methodologies in ornamental species breeding is dependent on information on the availability of efficient transformation and regeneration protocols, as well as the structure of plant genomes and function of genes. In recent decades genome sequencing technology played a significant role, allowing site-specific mutagenesis approaches on several key genes controlling traits of high interest and suggesting that CRISPR/Cas9-induced mutagenesis is effective also in ornamental sector (Zhang et al., 2016a; Kishi-Kaboshi et al., 2017; Yan et al., 2019; Yu et al., 2021b). In fact, it has been successfully employed to create gene knockouts and induce genetic alterations in ornamental *Petunia inflate* and *Petunia hybrid* (Subburaj et al., 2016; Zhang et al., 2016a; Sun and Kao, 2018; Yu et al., 2021b; Xu et al., 2022), *Chrysanthemum morifolium* (Kishi-Kaboshi et al., 2017), *Dendrobium officinale* (Kui et al., 2017), *Ipomoea nil* (Watanabe et al., 2017), *Lilium longiflorum* and *Lilium pumilum* (Yan et al., 2019), and *Phalaenopsis equestris* (Tong et al., 2020). In particular, in polyploid species, such as chrysanthemum, the possibility of mutating multiple copies of a target gene has been indirectly shown, as demonstrated in other polyploid crops, *e.g.*, hexaploid wheat (Wang et al., 2014; Mekapogu et al., 2022).

Regarding MS induction, the production of male-sterile ornamental plants is of great interest for many purposes, such as facilitating hybrid seed production, eliminating pollen allergens (i.e., gene escape), reduce the need for deadheading to extend the flowering period, redirect resources from seeds to vegetative growth and increase flower longevity and self-life (Garcia-Sogo et al., 2010). In past decades, the production of engineered male sterile plants by canonical transgenesis approaches was documented in ornamental *Kalanchoe blossfeldiana* through the directed expression of the ribonuclease Barnase gene under control of the PsEND1 promoter, which determines tissue-specific expression of the Barnase gene in anther tissues (epidermis, endothecium, middle layer, connective). The Barnase gene affected normal anther development, inducing the ablation of specific tissues at early stages of anther development with a consequent lack of pollen at anthesis in transgenic flowers (Garcia-Sogo et al., 2010). The use of this technology was especially useful to produce environmentally friendly transgenic ornamentals carrying new traits, as this modification would prevent gene flow between the genetically modified plants and related species (Roque et al., 2007; Gardner et al., 2009). A similar approach was used to efficiently create male sterile versions of existing *Pelargonium* spp. cultivars, which represent one of the most popular garden plants around the world, have considerable economic importance in the market of ornamental plants. Using a cotransformation protocol, two new traits were introduced in *P. zonale*, one to produce long-life plants by inducing the *IPT* gene during plant senescence and the other to produce male sterile plants without pollen (Garcia-Sogo et al., 2012). With similar molecular strategies and related purposes, male sterility was induced in C*hrysanthemums* spp. In this specific case, since many wild chrysanthemum relatives in the *Compositae* family are cross-compatible with chrysanthemum cultivars, to reduce the possibility of transgene flow into wild relatives, a male sterility trait using the mutated ethylene receptor gene Cm-ETR1/H69A was introduced into chrysanthemum cultivars (Shinoyama et al., 2012). Recently, thanks to the release of whole genome sequence information (Hirakawa et al., 2019), Shinoyama et al. (Shinoyama et al., 2020) reported an important example of MS induction in C*hrysanthemums* spp. by a genome editing approach targeting the *CmDMC1* gene through the use of TALENS technology to knock out all six identified *CmDMC1* genes. Two chrysanthemum cultivars with the TALEN expression vector resulted in the development of lines with disruption of all *CmDMC1* loci, successfully inducing male and female sterility (Shinoyama et al., 2020). The interest in creating MS lines in ornamental species, together with the positive results obtained in some of them, supports the idea of implementing CRISPR/Cas-based technologies as a potential tool for genetic improvement in floricultural research.

## 7 Concluding remarks and future perspective

Conventional breeding approaches still depend on breeders choosing materials based on phenotypic analyses. Breeders and scientists choose purposefully different parents to produce crop varieties that combine the desired characteristics of both parents. However, the usefulness of traditional breeding methods may be restricted to complex traits. To complement traditional breeding techniques, molecular breeders have developed and applied GE technologies, which should supplement rather than replace traditional breeding methods. Generally, two major criteria should be considered while assessing the applicability and future development of GE technology. Firstly, the development of cost-effective, low-risk, and efficient transformation systems that align with agricultural requirements is crucial in expanding the utilization of this molecular techniques. Secondly, the regulatory practices implemented by governments play a pivotal role. Currently, there is a global debate around whether CRISPR-edited lines should undergo similar regulations as conventional genetically modified (GM) plants, or if they should be allowed to enter the market without regulation once the CRISPR-cassette mediating segregating cycles have been removed (Chen and Gao, 2014; Voytas and Gao, 2014; Gao, 2015).

This review aims to emphasize that these approaches could make available potential and alternative methods for many breeding purposes. Several examples report that CRISPR/Cas technology has thus far been proven to be successful in genome editing of numerous food and non-food crops, as well as ornamental plants, whose genomes have been efficiently modified to induce genetic variability, resulting in a strong tool in plant genetics and precision breeding. The use of these modification tools, in comparison to their adaptability and final use, has provided a remarkable breakthrough in biological applications thanks to a growing number of accessible genome sequencing data related to the reduction in sequencing costs. In this intricate scenario, this review provides an overview of recent successes for MS induction based on GE applications, accelerating and lowering the cost of male sterility induction by targeting known candidate functional loci. The following development of male sterility, especially in food crops, has been greatly investigated for seed hybrid production. Conditional MS mutants, for example, created through genome editing, are particularly useful in major crops such as rice and maize, opening the possibility for applying the idea to many other crops Interestingly an increasing number of new additional applications of GE technology for MS producing have been reported, especially in promising ornamental species, in which the final goal of obtaining a MS ornamental species arises from the need to have allergenic free plant material. Furthermore, because only a few nucleotides are changed to modify the genome, the new improved methods based on simultaneous editing of gene sequences could be an important starting point for the development of new elite varieties by utilizing efficient and specific modifications at genomic loci, offering advantages over GM crops. Many of these new tools also benefit from TGE-based methods for editing additional alleles in polyploid species. Additionally, according to the studies mentioned above, the transient expression of the CRISPR/Cas cassette, and in particular the direct transfection of the RNP complex, exhibits a number of benefits compared to DNA plasmid delivery, followed by stable integration. With a transient approach, we have a DNA-free transfer, eliminating the possibility of unintended recombinant DNA insertion into the plant genome, a bypass of the cell’s transcriptional and translational machinery, with an immediate activity of the RNP complex per single cell, and finally a quick breakdown of complexes after delivery, which lowers the incidence of mosaicism effects. For these reasons, they are regarded as the most innovative and the new frontier of precision plant breeding programs, and this is the strategic direction that breeding could take in the future, supporting the idea that these approaches could be the new strategic assisted evolution technology towards reproductive systems, with potential to form new varieties.

## Author contributions

Conceptualization, SF and GB. Investigation and resources, SF, SD, and AB. Data curation, SF and FP. Writing—original draft preparation, SF, SD, and AB. Writing—review and editing, SF, FP, and AV. Visualization, SF, FP, AV, and GB. Supervision, ML and GB. Project administration, GB. Funding Acquisition, GB. All authors contributed to the article and approved the submitted version.
